# The implications of biomarker evidence for systematic reviews

**DOI:** 10.1186/1471-2288-12-176

**Published:** 2012-11-22

**Authors:** Miew Keen Choong, Guy Tsafnat

**Affiliations:** 1 Centre for Health Informatics, Australian Institute of Health Innovation, University of New South Wales, Sydney, Australia

**Keywords:** Biomarkers, Evidence-based medicine, Systematic review

## Abstract

**Background:**

In Evidence-Based Medicine, clinical practice guidelines and systematic reviews are crucial devices for medical practitioners in making clinical decision. Clinical practice guidelines are systematically developed statements to support health care decisions for specific circumstances whereas systematic reviews are summaries of evidence on clearly formulated clinical questions. Biomarkers are biological measurements (primarily molecular) that are used to diagnose, predict treatment outcomes and prognosticate disease and are increasingly used in randomized controlled trials (RCT).

**Methods:**

We search PubMed for systematic reviews, RCTs, case reports and non-systematic reviews with and without mentions of biomarkers between years 1990–2011. We compared the frequency and growth rate of biomarkers and non-biomarkers publications. We also compared the growth of the proportion of biomarker-based RCTs with the growth of the proportion of biomarker-based systematic reviews.

**Results:**

With 147,774 systematic reviews indexed in PubMed from 1990 to 2011 (accessed on 18/10/2012), only 4,431 (3%) are dedicated to biomarkers. The annual growth rate of biomarkers publications is consistently higher than non-biomarkers publications, showing the growth in biomarkers research. From 20 years of systematic review publications indexed in PubMed, we identified a bias in systematic reviews against the inclusion of biomarker-based RCTs.

**Conclusions:**

With the realisation of genome-based personalised medicine, biomarkers are becoming important for clinical decision making. The bias against the inclusion of biomarkers in systematic reviews leads to medical practitioners deprive of important information they require to address clinical questions. Sparse or weak evidence and lack of genetic training for systematic reviewers may contribute to this trend.

## Background

Evidence-Based Medicine (EBM) stipulates the use of the best available external scientific evidence in clinical decision making. Systematic Reviews (SRs) are conducted through a well defined process [1], and published as robust answers to clinical questions given the best available evidence [2]. Randomized Controlled Trials (RCTs) are trusted above all other primary evidence types [1]. EBM is thus implemented by following clinical practice guidelines (CPGs) or relying on SRs whenever possible when making clinical decisions. Guideline and SR development is a slow and complex process or translating research that can take more than a year [3] to complete. In this process it is critical to include all relevant evidence [4]. It was previously claimed that as much as 80% of all published reviews are not updated and only 3% of systematic reviews published in peer-reviewed journals had been updated within two years of their publication [5]. Only a small proportion of relevant trials are incorporated into systematic reviews [6].

The trend toward genome-based personalised medicine, and the rapid advancement of sequencing and high-throughput technologies, has dramatically reshaped disease research. Genomic data can now be obtained expeditiously and inexpensively. This has led to the use of a range of analytical tools to assess biological parameters [7].

Biomarkers that distinguish among subtypes of disease are now standard practice in biomedical research. There is an overwhelming interest in biomarker research reflected in a large number of research grants awarded and academic publications [8]. However, only a limited number of biomarkers have been incorporated into clinical guidelines [9], and the anticipation that biomarker research will revolutionize medical practice has so far not been realized. The ambiguity of the term “biomarker” has prompted the US National Institutes of Health (NIH) to define the terminology. Biomarkers are defined by the NIH Biomarkers Definitions Working Group as “a characteristic that is objectively measured and evaluated as an indicator of normal biological processes, pathogenic processes, or pharmacologic responses to a therapeutic intervention” [7]. Biomarkers are used as tools in disease diagnostic, early detection, staging, prognosis or prediction of treatment outcome. They can guide individualized treatment and improve patient care [9]. Biomarkers can provide the basis for design, improve the safety and efficiency, and explain empirical results of clinical trials [7].

The translation of biomarkers into clinical practice follows the regular translational pathways from discovery to preclinical, clinical, and post-approval trials and implementation. However, some translational crossroads are unique to biomarkers [10,11]. In particular, the diversity and lengthy process of biomarker assay development, and limited industry support were identified as bottlenecks for translating biomarker research into clinical practice. In this study, we examine the validation to implementation phase (also called T2 [12]). As there are cases of exaggeration in the effect sizes of many highly cited biomarkers studies (which lead to overestimated findings) [13], we measured the inclusion of RCTs of biomarkers in SRs. We believe that prospective evaluations of biomarkers in RCTs could provide more reliable results about their effects and clinical utility.

We address the following questions:

1. How does the output frequency of publications that include biomarkers compare to those that don’t?

2. Is the growth rate of publication of biomarkers RCTs in line with the non-biomarkers publications?

3. To which extent biomarkers RCTs are being included in SRs as compared to non-biomarkers RCTs?

## Methods

Although there is no single database reliably showing the true number of SRs or RCTs [6], we used PubMed as the basis of our search. We searched for systematic reviews, controlled trials, case reports and non-systematic reviews by using the publication type in PubMed's Entrez search engine using the terms shown in Table 1.

**Table 1:** Publication type search based on publication types of PubMed

**Classification**	**Publication type in PubMed**
Systematic reviews (SR/MA)	“Systematic Reviews” OR “Meta-Analysis”
Controlled Trial (RCT/CCT)	“Randomized Controlled Trial” OR “Controlled Clinical Trial”
Case Reports	“Case Reports”
Non-systematic reviews	“Review”

There are ambiguities in the naming and terminology of the term “biomarker” within the literature [8]. As we are interested in all biomarkers, we have included as many terms with overlapping meaning of “biomarker” as possible, such as biological marker/s, molecular marker/s, genetic marker/s, DNA marker/s, cytogenetic marker/s, proteomics marker/s and biochemical marker/s. We repeated each search with (biomarker OR "biological marker" OR “biological markers” OR "molecular marker" OR "molecular markers" OR "genetic marker" OR "genetic markers" OR "DNA marker" OR "DNA markers" OR "cytogenetic marker" OR "cytogenetic markers" OR "proteomics marker" OR "proteomics markers" OR "biochemical marker" OR "biochemical markers") helped restrict the results to those biomarkers publications only, and (all[sb] NOT (biomarker OR "biological marker" OR “biological markers” OR "molecular marker" OR "molecular markers" OR "genetic marker" OR "genetic markers" OR "DNA marker" OR "DNA markers" OR "cytogenetic marker" OR "cytogenetic markers" OR "proteomics marker" OR "proteomics markers" OR "biochemical marker" OR "biochemical markers")) for non-biomarkers publications. “All publications” is the summation of the above two complementary groups. We limited our search to human subjects and to documents in English. All searches were done on the 18^th^ of October 2012.

In order to validate the search strategy, we randomly chose 110 search results from each group (biomarkers and non-biomarkers) of RCT/CCT (“Randomized Controlled Trial” or “Controlled Clinical Trial”) and manually read their abstracts and if needed their full text, to determine if the trial is indeed about biomarkers or using biomarkers as outcome measures.

The average annual growth rates are calculated using the following equation:

$$\frac{1}{N}\overset{N}{\underset{i=2}{\sum }}\frac{x_{i}-x_{i-1}}{x_{i-1}}$$

where *x*_*i*_ is the current year's total publications and *N*=*22* is the total number of years included in the study.

In order to assess the extent of inclusions of clinical trials publications in systematic reviews, we normalized the systematic reviews samples by the number of published clinical trials.

## Results

The validation of search results on RCT/CCT yields an accuracy of 0.82, with sensitivity of 0.76 and specificity of 0.94.

While only a minority of trials has been included in systematic reviews [6], this study found that the number of trials involving biomarkers assessed in systematic reviews is even smaller. With 147,774 systematic reviews indexed in PubMed from 1990 to 2011 (accessed on 18/10/2012), only 4,431 (3%) are dedicated to biomarkers.

**Figure 1:**
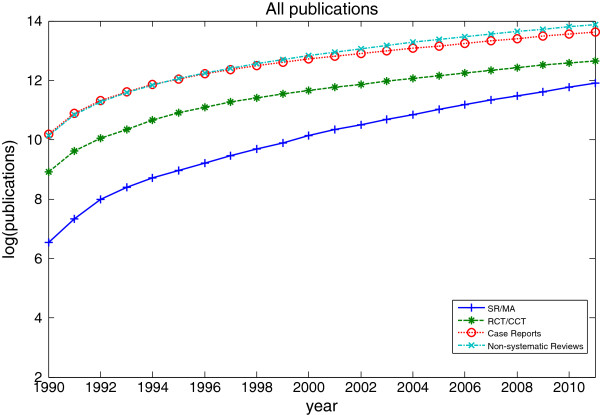
**The cumulative number of all published systematic reviews, controlled trials, case reports and non-systematic reviews.** The cumulative number of all published systematic reviews, controlled trials, case reports and non-systematic reviews as identified in PubMed, 1990 to 2011.

Figure 1 shows a logarithmic representation of the cumulative number of publications of clinical evidence (SR/MT (“Systematic Reviews” or “Meta-Analysis”), RCT/CCT, case reports and non-systematic reviews). The growth of the publications of clinical evidence appears to support the Prices Law of exponential growth [14]. The total number of publications has increased tremendously, from as low as 691 for SR/MA, 7402 for RCT/CCT, 26,721 for case reports, and 25,261 for non-systematic reviews in 1990 to 19,195, 23,488, 51,019, 74,411 in year 2011 respectively.

**Figure 2:**
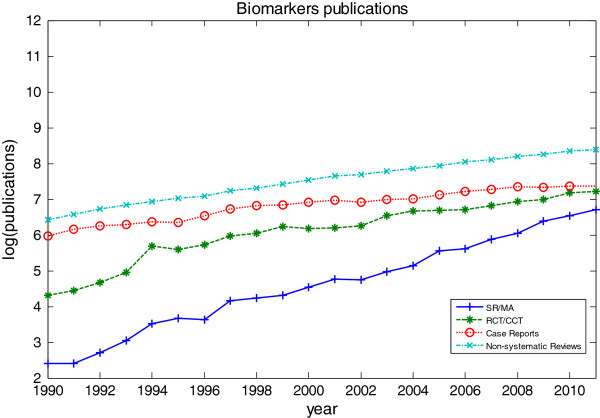
**The number of published systematic reviews, controlled trials, case reports and non-systematic reviews on biomarkers on a logarithmic scale.** The number of published systematic reviews, controlled trials, case reports and non-systematic reviews on biomarkers as identified in PubMed, 1990 to 2011.

**Figure 3:**
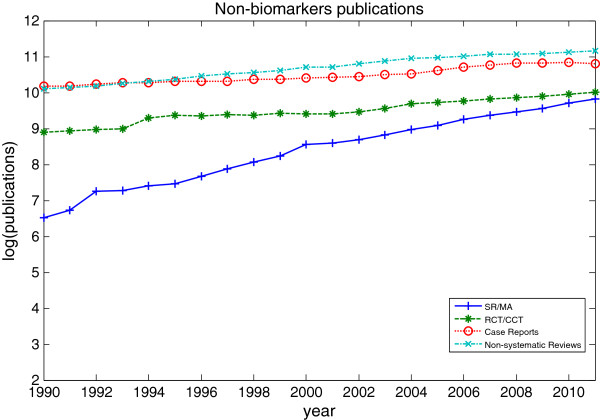
**The number of published systematic reviews, controlled trials, case reports and non-systematic reviews on non-biomarkers on logarithmic scale.** The number of published systematic reviews, controlled trials, case reports and non-systematic reviews on non-biomarkers as identified in PubMed, 1990 to 2011.

Figure 2 and Figure 3 show a logarithmic representation of the total number of publications of SR/MA, RCT/CCT, case reports and non-systematic reviews on biomarkers and non-biomarkers from 1990 to 2011. Figure 2 shows that as the number of published trials, case reports and non-systematic reviews increased for biomarkers publications, so did the number of systematic reviews. Figure 3 shows the same trend for non-biomarker publications. The average annual growth rates of biomarkers and non-biomarkers publications are shown in Table 2. The annual growth rate of case reports and non-systematic reviews are comparatively lower than the SR/MA and RCT/CCT which are at the top two of the hierarchy of clinical evidence. As evidenced by annual growth rate where biomarkers publications are consistently higher than non-biomarkers publications, there is a growth in biomarkers research [15].

**Table 2:** Total publications and average annual growth rates of biomarkers and non-biomarkers publications from year 1990-2011

	**Biomarkers**		**Non-biomarkers**	
	**Total publications (1990–2011)**	**Annual growth rate (%)**	**Total publications (1990–2011)**	**Annual growth rate (%)**
SR/MA	4431	24.25	143343	17.67
RCT/CCT	12866	16.68	302940	5.64
Case Reports	22005	7.14	798765	3.09
Non-systematic reviews	47436	9.85	1014886	5.13

**Figure 4:**
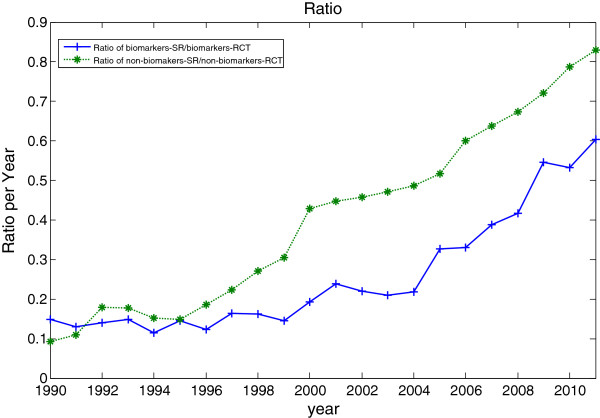
**Comparison of ratio of systematic reviews over controlled trials for biomarkers and non-biomarkers publications.** Comparison of ratio of systematic reviews over controlled trials for biomarkers and non-biomarkers publications, 1990 to 2011.

The ratio of systematic reviews over the published clinical trials (ratio SR/RCT) of biomarkers and non-biomarkers are shown in the solid line and dotted line of Figure 4 respectively. The ratio SR/RCT represents how many systematic reviews are published per clinical trials in the same year. In other words, how likely a published clinical trial is to be included in a SR. The ration SR/RCT of both biomarkers and non-biomarkers publications is growing, reflecting the efforts of the review community in assessing the published trials and producing systematic reviews at a growing rate. The ratio SR/RCT of non-biomarkers publications is growing and reached over 0.8, but the increased ratio SR/RCT on biomarkers is comparatively lower and only reached 0.6 (if following the trend of overall publication, there are about 20% to over 100% less systematic reviews on biomarkers over the 22 years). The results show that there are fewer systematic reviews published per published clinical trials in biomarker-domain.

**Figure 5:**
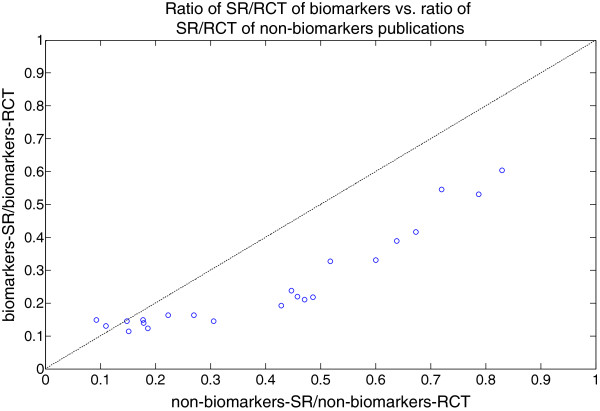
**Ratio of SR/RCT of biomarkers vs. ratio of SR/RCT of non-biomarkers publications.** Ratio of SR/RCT of biomarkers vs. ratio of SR/RCT of non-biomarkers publications, 1990 to 2011.

Figure 5 is a plot of ratio SR/RCT of biomarkers versus SR/RCT of non-biomarkers publications. The dotted line in Figure 5 represents the equal ratio of biomarkers and non-biomarkers publications trend. The biomarkers SR/RCT is consistently below that of non-biomarker publications as shown in Figure 4 and Figure 5. With the hypothesis that the likelihood of published clinical trials is being included in systematic reviews for biomarkers and non-biomarkers are the same, it is clear that there is a preference towards non-biomarkers publications.

**Figure 6:**
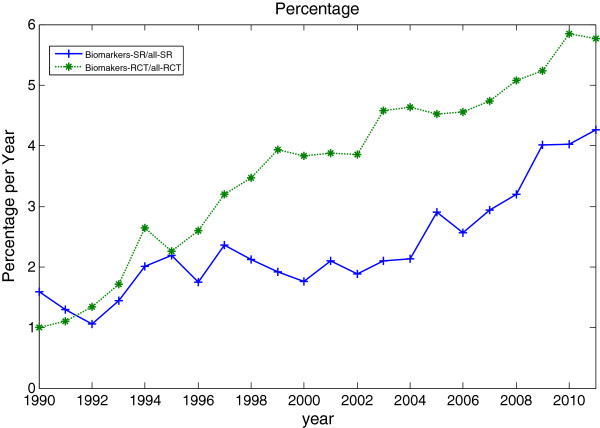
**Comparison of percentages of clinical trials and systematic reviews on biomarkers.** Comparison of percentage of clinical trials of biomarkers over all clinical trials and percentage of systematic reviews on biomarkers over all systematic reviews as identified in PubMed, 1990 to 2011.

From Figure 6, we can observe that the percentages biomarkers publications in clinical trials and systematic reviews are increasing. However, the proportion increase in biomarker systematic reviews is comparatively lower (the percentages in early 1990s might not be a good indication as there are only a low number of published trials and systematic reviews). Figure 6 also strengthen the evidence that biomarkers are an active field of research as the proportion of both biomarkers trials and systematic reviews increases. The growth in the number of biomarkers used in clinical trials and systematic reviews has shown its importance.

## Discussion

Biomedical research articles often include poor reporting of statistical methods. Incomplete reporting of statistical analysis limits or prevents the use of these studies in the systematic reviews. This could explain why only a small proportion of RCTs are included in systematic reviews. Further study is needed to test if biomarkers RCTs are more prone to bad statistical methods reporting than other RCTs.

Our results show discrepancy between the rate of publications of biomarkers RCTs and biomarkers reviews. Possible explanations for this include:

• The frequent proposals of new biomarkers and assays which have complicated the translation and commercialization processes [9].

• Independent investigations in biomarkers show contradicting results. A biomarker is ready for clinical testing only when several retrospective tests consistently confirm its performance [10]. As such, weak and sparse evidence could not lead to any conclusion.

• Systematic reviewers shy away from a domain in which their training is lacking [16].

• Genetic disorders, where specific genetic variants causally associated with common diseases, account for only a fraction of cases [17], which result in smaller cohorts for studies.

• RCTs, observational studies or cross-sectional diagnostic studies are designed to answer generalized clinical questions and ignore outliers. Biomarker evidence might be too sensitive to outliers [18] to provide low p-values.

• A few outliers might also cause small effects even in large cohort studies [19] which would result in exclusion from systematic reviews as they will provide no clear difference from previous summarized evidence.

• Observational studies often focus on statistical significance which doesn't guarantee high reproducibility [20,21].

As systematic reviews are the main source for guideline development, the lack of effort on systematically reviewing studies in this area can explain why only a limited number of molecular markers have been incorporated into clinical guidelines. There are still many relevant trials not being assessed or not included in systematic reviews and guidelines. This seems to be stronger in biomarker evidence as we have shown.

## Conclusions

Biomarkers are becoming increasingly valuable in clinical settings, whether to diagnose, prognosticate or to guide treatment. It is important to fast track the research and translation process. We identified the need for systematic reviewers to include more biomarkers and proposed several possible explanations of why this has not been done yet including lacking education of systematic reviewers on molecular biology concepts and the low predictive power of biomarkers. We propose that specific search technologies can support the review process.

## Competing interests

The authors declare that they have no competing interests.

## Authors' contributions

MKC conceived the study, gathered and analysed data, and drafted the manuscript. GT conceived the study, interpreted the information, and drafted the manuscript. Both authors read and approved the final manuscript.

## Pre-publication history

The pre-publication history for this paper can be accessed here:

http://www.biomedcentral.com/1471-2288/12/176/prepub
